# Vascular pedicle width in acute lung injury: correlation with intravascular pressures and ability to discriminate fluid status

**DOI:** 10.1186/cc10084

**Published:** 2011-03-07

**Authors:** Todd W Rice, Lorraine B Ware, Edward F Haponik, Caroline Chiles, Arthur P Wheeler, Gordon R Bernard, Jay S Steingrub, R Duncan Hite, Michael A Matthay, Patrick Wright, E Wesley Ely

**Affiliations:** 1 Division of Allergy, Pulmonary, and Critical Care Medicine, Vanderbilt University School of Medicine, T-1218 MCN Nashville, TN 37221, USA; 2 Section on Pulmonary, Critical Care, Allergy, and Immunologic Diseases, Wake Forest University School of Medicine, Medical Center Blvd, Winston-Salem, NC 27157, USA; 3 Division of Radiological Sciences, Department of Radiology, Wake Forest University School of Medicine, Medical Center Blvd, Winston-Salem, NC 27157, USA; 4 Division of Critical Care Medicine, Baystate Medical Center, 759 Chestnut St, Springfield, MA 01199, USA; 5 Department of Medicine and Anesthesia, Cardiovascular Research Institute, University of California, San Francisco, 505 Parnassus Avenue, Moffitt Hospital, M-917, San Francisco, CA 94143, USA; 6 Department of Pulmonary and Critical Care Medicine, Moses Cone Health System, 1200 N Elm St, Greensboro, NC 27403, USA

## Abstract

**Introduction:**

Conservative fluid management in patients with acute lung injury (ALI) increases time alive and free from mechanical ventilation. Vascular pedicle width (VPW) is a non-invasive measurement of intravascular volume status. The VPW was studied in ALI patients to determine the correlation between VPW and intravascular pressure measurements and whether VPW could predict fluid status.

**Methods:**

This retrospective cohort study involved 152 patients with ALI enrolled in the Fluid and Catheter Treatment Trial (FACTT) from five NHLBI ARDS (Acute Respiratory Distress Syndrome) Network sites. VPW and central venous pressure (CVP) or pulmonary artery occlusion pressure (PAOP) from the first four study days were correlated. The relationships between VPW, positive end-expiratory pressure (PEEP), cumulative fluid balance, and PAOP were also evaluated. Receiver operator characteristic (ROC) curves were used to determine the ability of VPW to detect PAOP <8 mmHg and PAOP ≥18 mm Hg.

**Results:**

A total of 71 and 152 patients provided 118 and 276 paired VPW/PAOP and VPW/CVP measurements, respectively. VPW correlated with PAOP (r = 0.41; *P *< 0.001) and less well with CVP (r = 0.21; *P *= 0.001). In linear regression, VPW correlated with PAOP 1.5-fold better than cumulative fluid balance and 2.5-fold better than PEEP. VPW discriminated achievement of PAOP <8 mm Hg (AUC = 0.73; *P *= 0.04) with VPW ≤67 mm demonstrating 71% sensitivity (95% CI 30 to 95%) and 68% specificity (95% CI 59 to 75%). For discriminating a hydrostatic component of the edema (that is, PAOP ≥18 mm Hg), VPW ≥72 mm demonstrated 61.4% sensitivity (95% CI 47 to 74%) and 61% specificity (49 to 71%) (area under the curve (AUC) 0.69; *P *= 0.001).

**Conclusions:**

VPW correlates with PAOP better than CVP in patients with ALI. Due to its only moderate sensitivity and specificity, the ability of VPW to discriminate fluid status in patients with acute lung injury is limited and should only be considered when intravascular pressures are unavailable.

## Introduction

The NIH NHLBI ARDS Network Fluid and Catheter Treatment Trial (FACTT) demonstrated that fluid management for patients with acute lung injury (ALI) using a protocol guided by intravascular pressure measurements from a central venous catheter (CVC) resulted in similar clinical outcomes compared to fluid management directed by measurements from a pulmonary artery catheter (PAC) [1]. The PAC group experienced significantly more nonfatal complications, mostly in the form of arrhythmias. These results, combined with previous studies demonstrating either lack of benefit or increased harm, have led many experts to discourage the routine use of the PAC in patients with ALI [2,3]. Regardless of the type of catheter, a conservative fluid management strategy in ALI patients increased the number of days alive and free from mechanical ventilation [4]. Central venous pressure (CVP) or pulmonary artery occlusion pressure (PAOP) was used to generate instructions and function as targets for the fluid management strategies in this trial. It remains unknown if such invasive measurements are required for management of critically ill patients or if non-invasive measurements would suffice.

Portable chest x-rays (CXR) are obtained frequently in patients with ALI. In previous studies, the vascular pedicle width (VPW), either alone or in conjunction with the cardiothoracic ratio (CTR), which are both easily measured on most portable CXRs [5], has correlated with intravascular volume status in both critically ill and non-critically ill patients [6-11]. Despite these data, monitoring of VPW is not part of standard practice. The purpose of this study was to investigate the relationship between non-invasive measures of intravascular volume status, namely the VPW and CTR and invasive intravascular pressure measurements, namely CVP and/or PAOP, in ALI patients enrolled in the FACTT study at five Acute Respiratory Distress Syndrome (ARDS) Network sites. In addition, the ability of VPW to discriminate when the edema had a hydrostatic component or when conservative fluid management goals were achieved was also investigated.

## Materials and methods

Patients included in this analysis were a subset of patients enrolled in the ARDS Network Fluid and Catheter Treatment Trial (FACTT). All centers enrolling in FACTT obtained local IRB approval and all patients or their surrogates provided informed consent. This data analysis was also specifically considered exempt by the Vanderbilt Institutional Review Board. FACTT was a multi-center, randomized clinical trial of two different fluid strategies (conservative vs. liberal) factorialized with two different methods of intravascular pressure measurement (CVP or PAOP). The patients randomized to receive PAC had both PAOP and CVP measurements while only CVP measurements were available for those randomized to management with a CVC. Neither CVP nor PAOP measurements were adjusted for positive-end expiratory pressure (PEEP) levels. FACTT used a standardized fluid management protocol [4], which attempted to achieve intravascular pressure targets when patients were not in shock and had adequate renal and circulatory function. Intravascular pressure measurements were taken every four hours for the shorter of seven days or duration of mechanical ventilation. Two intravascular measurements were recorded daily; one from 08:00 AM and a second from a random protocol check time which changed each day. To be eligible for this substudy, patients enrolled in FACTT must have had both a chest radiograph available for review and a "matching" intravascular pressure measurement for any day between study days 0 through 4. Matching intravascular pressure measurement was defined as a CVP and/or PAOP measurement obtained within three hours before or after the time of the chest radiograph. In the case of two recorded intravascular pressure measurements within the desired time window, the one closest to the time of the CXR was used. When two CXRs within the time window for a single pressure measurement were available, the closest CXR was utilized.

### Chest radiograph interpretation

De-identified digital copies of the chest radiographs were sent to Vanderbilt for central distribution to the readers. In instances where the CXR was not available in digital format, de-identified hard copies were utilized. All radiographs were interpreted independently by five investigators; a radiologist (CC), two intensivists experienced at measuring VPW (EWE, EH), and two intensivists inexperienced at measuring VPW (TWR, LBW). The inexperienced intensivists received a half day training session reading VPW and CTR measurements alongside an experienced intensivist prior to interpreting the films for this study. The radiographs were scored by each reader as satisfactory or unsatisfactory with regard to both positioning and technique. At least three of the five readers had to score the radiograph as satisfactory for both positioning and technique in order for the measurements to be utilized in the final analysis. Each reader also independently measured the VPW and CTR (see below) for each radiograph that they scored as satisfactory for both positioning and technique. The VPW and CTR values were averaged to obtain a single VPW and CTR measurement for each radiograph. All of the roentgenographic interpretations were performed in a blinded fashion.

### Vascular pedicle width and cardiothoracic ratio measurements

**Figure 1:**
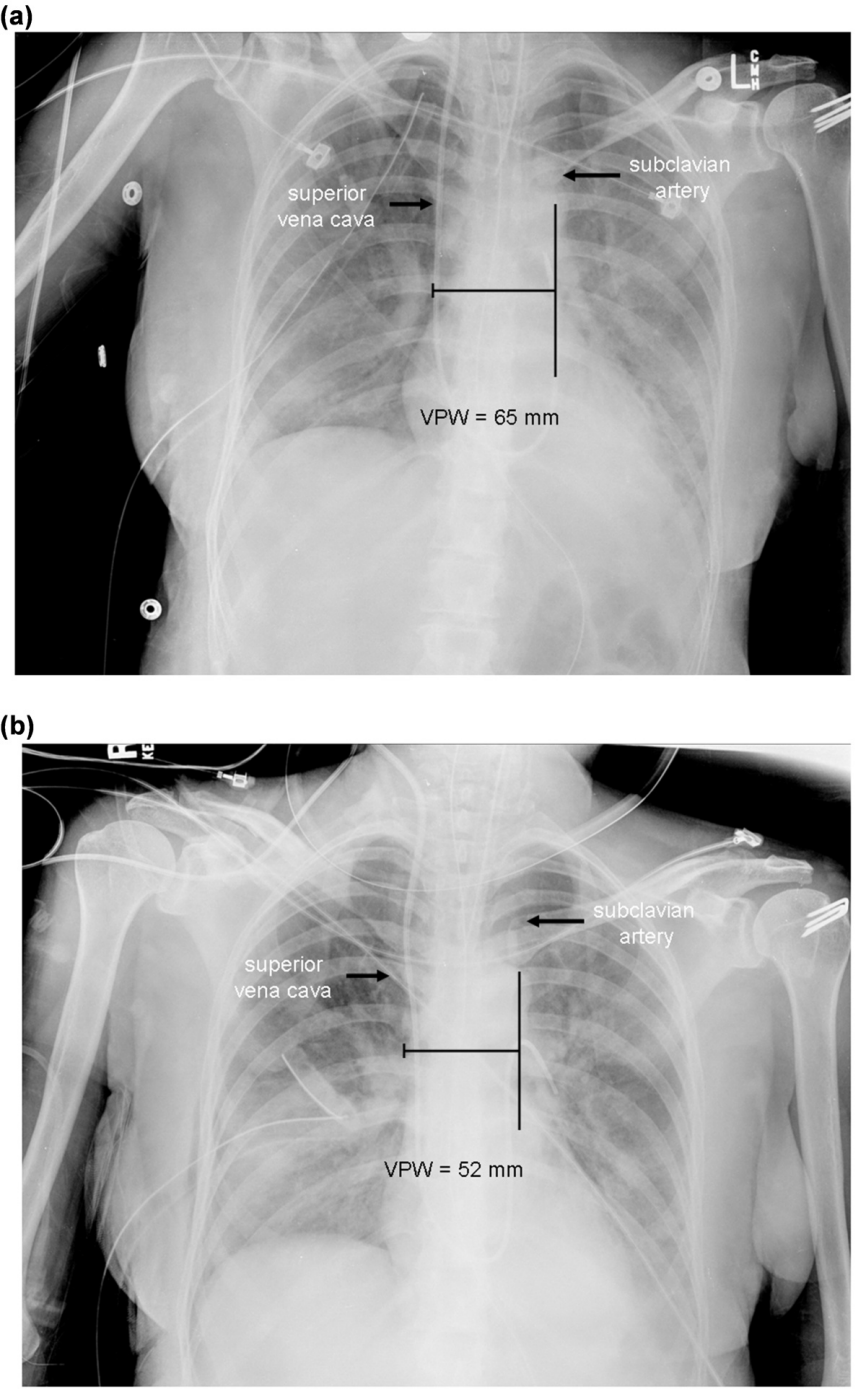
**Representation of the VPW measurement and change in VPW over time**. The VPW is the distance between where the left subclavian artery exits the aortic arch and where the superior vena cava crosses the right mainstem bronchus. **(a-b) **represent CXRs from the same patient at baseline and Day 3, respectively, where the VPW has decreased by 13 mm.

The vascular pedicle width represents the mediastinal silhouette of the great vessels. First described in detail by Milne and colleagues two decades ago, VPW is the distance from which the left subclavian artery exits the aortic arch measured across to the point at which the superior vena cava crosses the right mainstem bronchus (Figure 1) [5]. The vertical lateral border of the superior vena cava or right brachiocephalic vein was utilized for the measurement in radiographs where the right border of the vascular pedicle was indistinct. The cardiothoracic ratio was calculated by dividing the measurement of the largest width of the cardiac silhouette by the interior width of the thoracic cavity at the same vertical location.

### Covariates

A number of covariates were collected prospectively during the FACTT trial that may also have influenced both VPW and/or the intravascular pressure measurements (Table 1). Net fluid balance was collected for the 24 hours prior to enrollment and then every day until the earlier of extubation, death, or study Day 7. PEEP was recorded from morning ventilator measurements daily through study Day 7. Serum albumin was measured at baseline.

**Table 1:** Multivariate regression of VPW, net fluid balance, PEEP, and albumin with PAOP

	Unstandardized coefficients		95% CI for B		Standardized coefficients	*P*-value
				
	B	Std. error	Lower bound	Upper bound		
**Constant**	**-3.34**	**4.05**	**-11.39**	**4.71**		**0.41**
**VPW**	**0.20**	**0.04**	**0.11**	**0.29**	**0.43**	**<0.001**
**Cumulative Net Fluid (L)**	**0.21**	**0.08**	**0.05**	**0.37**	**0.26**	**0.01**
**PEEP**	**0.26**	**0.14**	**-0.02**	**0.54**	**0.19**	**0.07**
**Albumin**	**1.05**	**0.90**	**-0.73**	**2.84**	**0.11**	**0.24**

Standardized coefficients allow comparison of the covariate correlations to PAOP. For example, VPW correlates with PAOP about 2.5 times as well as PEEP (0.42 vs. 0.19). 95% CI, 95% confidence interval; VPW, vascular pedicle width; PEEP, positive end-expiratory pressure; PAOP, pulmonary artery occlusion pressure.

### Statistical analysis

Correlation between VPW measurements from the portable chest radiograph with the PAOP represented the primary endpoint. Secondary endpoints included correlation of VPW and CTR with both PAOP and CVP. The effect of cumulative fluid balance, PEEP, and serum albumin on the relationship between VPW and PAOP represented additional secondary endpoints. A formal sample size calculation was not undertaken as this study utilized all available patients with matching CXR and vascular pressure measurements from the five sites. The mean VPW and CTR were determined for each individual radiograph by averaging the measurements from all the readers who gave a satisfactory grade to position and technique for that radiograph. Inter-rater variability was assessed by calculating the difference between readings for each pair of readers for each measurement. These differences were then averaged and divided by the mean value of the reading to obtain the relative percent variation. VPW and CTR were compared separately to both CVP and PAOP measurements using scatterplots with regression equations. R values were determined using Spearman's correlations. Multivariate linear regression analysis was utilized to determine the effect that cumulative fluid balance, PEEP, and baseline serum albumin had on the relationship between VPW and PAOP. All variables were included in the model regardless of the significance of their association. Both the net fluid balance for the day of the intravascular pressure measurement and the cumulative net fluid balance from 24 hours prior to enrollment through the day of the VPW measurement were included in the multivariate regression analysis separately. Standardized coefficients were obtained to compare the relative effect each covariate had on PAOP. Cumulative net fluid balance from 24 hours prior to enrollment through the day of the VPW measurement had a better correlation than the daily fluid balance, so it was utilized in the final model. The PEEP value used in the regression analysis was the morning (that is, 06:00 to 10:00 AM) value from the day of the CXR. Receiver operating characteristic (ROC) curves were utilized to determine both the optimal VPW cutoff for discriminating adequateness of conservative fluid management, defined as a PAOP measurement <8 mmHg and whether some component of hydrostatic edema may also be present (that is, PAOP ≥18 mm Hg). Sensitivity, specificity, and likelihood ratios of the VPW cutoff value were calculated using Confidence Interval Analysis 2.1.0 [12]. The change in VPW over time was calculated from the first CXR to the last available CXR in patients with two CXRs at least 48 hours apart between baseline and study Day 4. The median change in VPW over time was compared between conservative and liberal treatment strategy groups using Mann Whitney U testing. Data were analyzed using SPSS (Version 15.0; Chicago, IL, USA) and two-sided *P*-values ≤0.05 were utilized to determine statistical significance.

## Results

**Figure 2:**
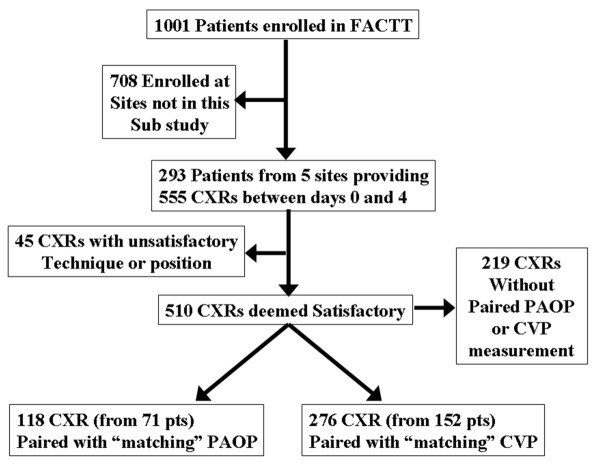
**Flow diagram showing study enrollment and available CXRs**.

Of the 1,001 patients enrolled in FACTT, 293 were enrolled at one of the five sites participating in this study. Those 293 patients provided 555 CXRs through study Day 4 for interpretation. Of the available 555 CXRs, 510 (91.9%) were deemed satisfactory for both technique and position by at least three of the reviewers. Of the satisfactory CXRs, 118 (from 71 patients) were able to be paired with a "matching" PAOP measurement (that is, within three hours of the measurement) and 276 (from 152 patients) were able to be paired with a "matching" CVP measurement (Figure 2). The average CVP and PAOP for the paired measurements were 11.9 ± 5.1 and 16.2 ± 5.4 mmHg, respectively. In the 118 pairs with both measurements available, PAOP and CVP were highly correlated (CVP = 0.58 + 0.73*PAOP; r = 0.74; *P *< 0.001). The average VPW and CTR for paired measurements was 71.8 ± 11.2 mm and 0.56 ± 0.06, respectively. The correlation between VPW and CTR (r = 0.33; *P *< 0.001) was also significant, but less strong than that between PAOP and CVP. The average difference between readers' measurements were 8 ± 6 mm for cardiac width, 6 ± 5 mm for thoracic width, and 8 ± 4 mm for VPW. These represent relative percent variations of 5 ± 4%, 2 ± 2%, and 11 ± 6%, for cardiac, thoracic, and VPW measurements, respectively.

### VPW, CTR, and intravascular pressure measurement correlations

**Figure 3:**
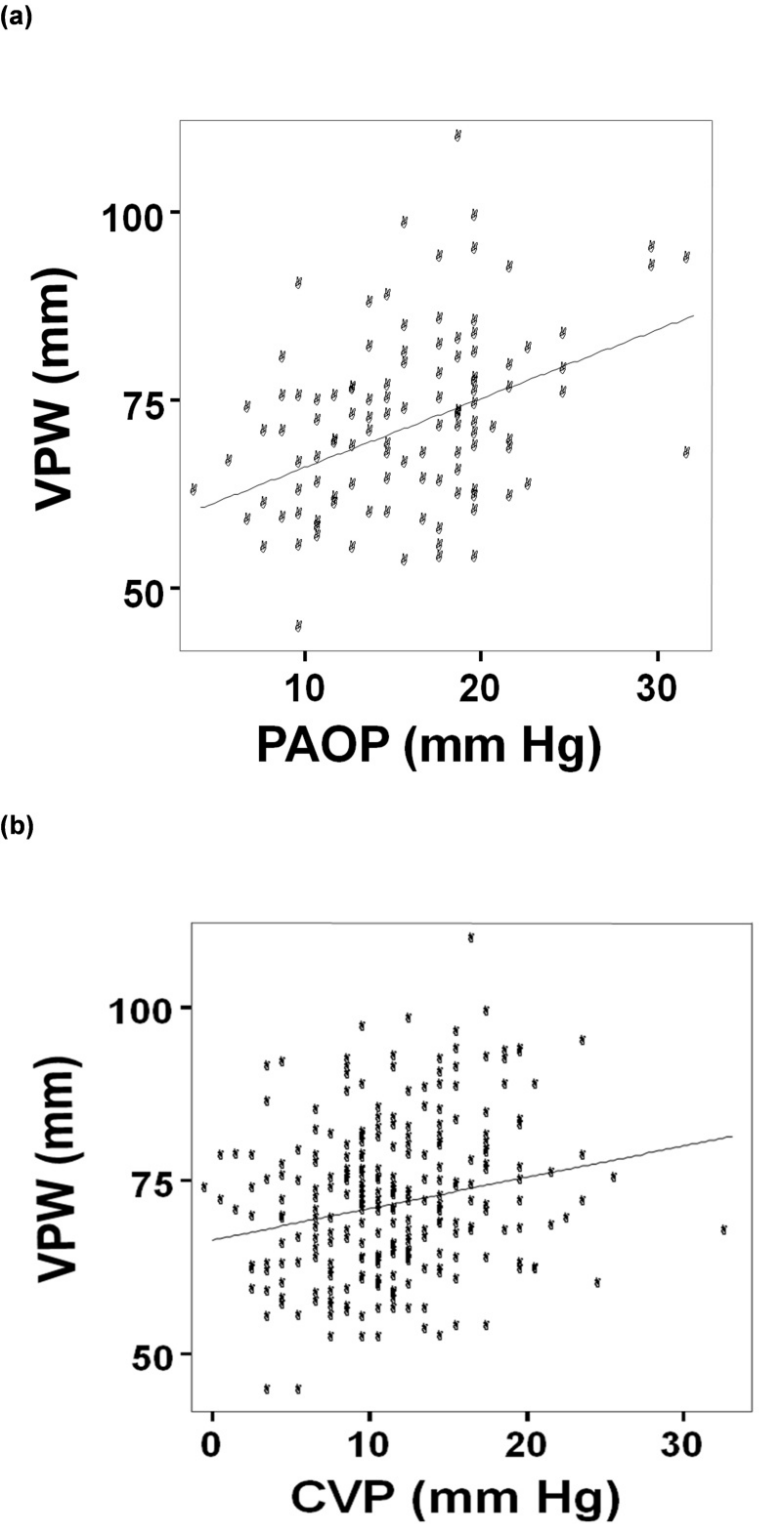
**Correlation of VPW with PAOP and CVP**. **(a) **demonstrates that VPW correlates moderately well with PAOP (VPW = 57 + 0.9*PAOP; r = 0.41; *P *< 0.001). **(b) **demonstrates the weak correlation between VPW and CVP (VPW = 66.4 + 0.45*CVP; r = 0.21; *P *= 0.001).

The VPW decreased by a median width of 1.8 (interquartile range (IQR): -7.2 to + 3.5) mm over time in patients assigned to the conservative (n = 72) fluid management strategy compared to a median increase in width of 2.3 (IQR: -4.4 to +8.8) mm in those assigned to the liberal fluid management strategy (n = 77) (*P *= 0.012). For these same patients, conservative fluid management strategy resulted in a less positive cumulative fluid balance (742 ± 7,986 vs. 6,553 ± 7,913 cc; *P *< 0.001). Figure 3a shows a scatterplot demonstrating the relationship between VPW and PAOP while Figure 3b demonstrates the relationship between VPW and CVP. Although statistically significant, VPW did not highly correlate with either PAOP (r = 0.41; *P *< 0.001) or CVP (r = 0.21; *P *= 0.001). The relationship between VPW and PAOP is described by the linear regression equation: VPW = 57 + 0.9*(PAOP) while the equation: VPW = 66.4 + 0.45*(CVP) describes the correlation with CVP. Cardiothoracic ratio correlated modestly with PAOP (r = 0.30; *P *= 0.001) and demonstrated little correlation with CVP (r = 0.15; *P *= 0.01).

### VPW, PAOP and covariates

PAOP was positively correlated with VPW (r = 0.41; *P *< 0.001), cumulative net fluid balance to the time of the paired measurement (r = 0.31; *P *= 0.002), and PEEP (r = 0.22; *P *= 0.02) but not serum albumin (*P *= 0.23). VPW did not correlate significantly with cumulative fluid balance (*P *= 0.46), PEEP (*P *= 0.21), or serum albumin (*P *= 0.20). Multivariate regression analysis demonstrated that VPW and cumulative fluid balance independently correlated with PAOP and PEEP trended toward a correlation with PAOP. Serum albumin did not correlate with VPW in multivariate analysis. Standardized coefficients indicate that VPW had a 1.5-fold stronger correlation with PAOP than cumulative fluid balance and a 2.5-fold stronger correlation than PEEP (Table 1).

### Optimal VPW for discriminating adequacy of conservative fluid management or hydrostatic component to the edema

**Figure 4:**
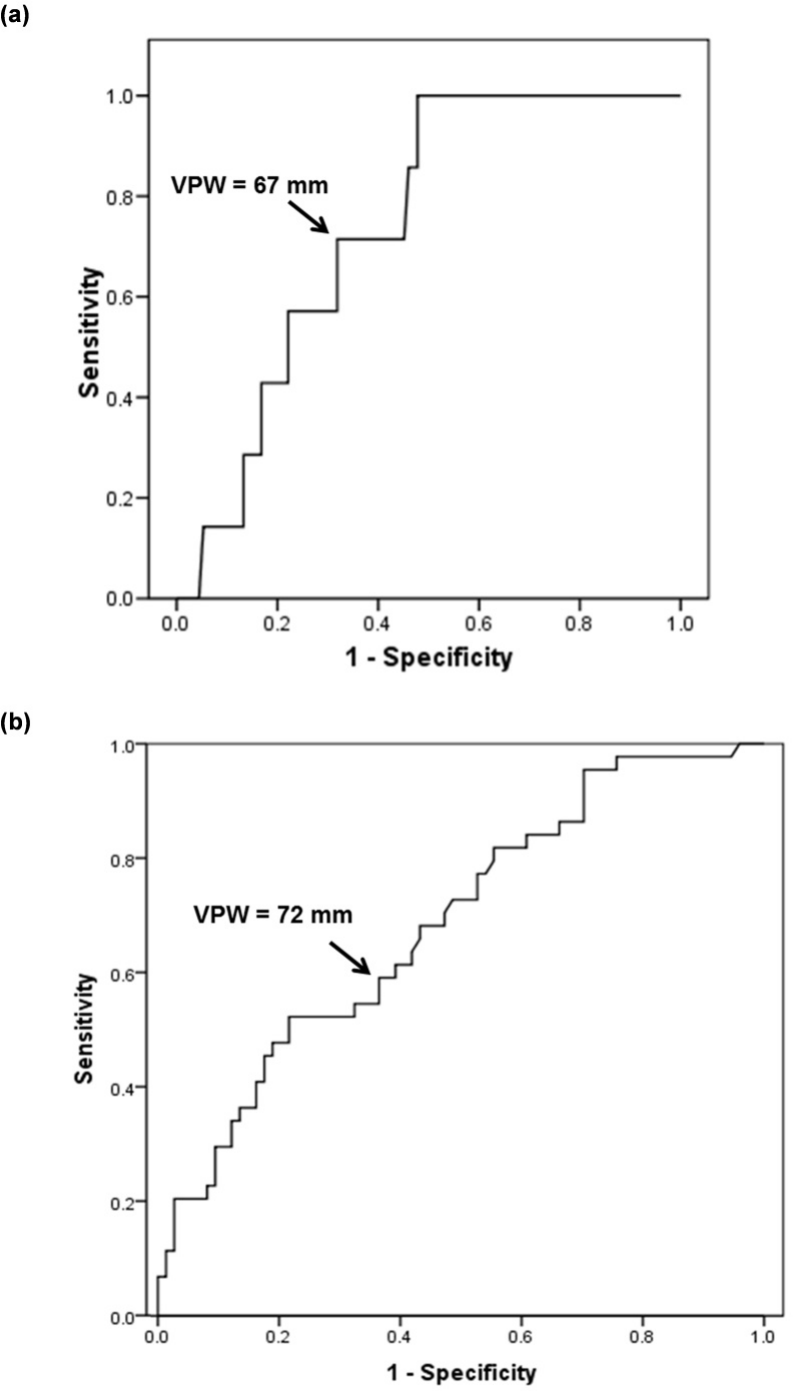
**ROC curve for VPW discriminating fluid status by PAOP**. **(a) **demonstrates that VPW of 67 mm discriminates PAOP <8 mmHg (AUC = 0.73; *P *= 0.04). **(b) **demonstrates that VPW of 72 discriminates PAOP ≥18 mmHg (AUC = 0.69; *P *= 0.001).

Only seven (6%) of the 118 PAOP and 19 (7%) of the 276 CVP measurements were within the target range for conservative fluid management strategy (that is, PAOP <8 or CVP <4 mm Hg). The ROC curve (Figure 4a) demonstrates the ability of VPW to discriminate achieving PAOP <8 mm Hg (AUC = 0.73; 95% CI: 0.59 to 0.87; *P *= 0.04). A VPW ≤67 mm had 71.4% sensitivity (95% CI 30.1 to 95.4%) and 67.6% specificity (95% CI 58.5 to 75.4%) for predicting PAOP <8 mm Hg. Due to the high percentage of measurements outside the target range, however, a VPW greater than 67 mm had a negative predictive value of 97.4% (95% CI 91.0 to 99.3%) for PAOP ≥8 mm Hg. The positive and negative likelihood ratios for the VPW cutoff of 67 mm discriminating PAOP <8 (that is, conservative fluid strategy target range) were 2.2 (95% CI: 1.3 to 3.8) and 0.42 (95% CI: 0.13 to 1.3), respectively. VPW was not able to discriminate achieving the conservative fluid management target using CVP (that is, CVP <4 mmHg) (AUC = 0.57; 95% CI: 0.43 to 0.70; *P *= 0.32).

Over a third (44/118) of the PAOP measurements were ≥18 mm Hg, suggesting a hydrostatic component to the edema in these patients with lung injury. A VPW cutoff ≥72 mm best discriminated a PAOP ≥18 mm Hg (AUC 0.686; 95% CI 0.589 to 0.784; *P *= 0.001) (Figure 4b). This cutoff demonstrated 61.4% sensitivity (95% CI 46.6 to 74.3%) and 60.8% specificity (95% CI 49.4 to 71.1%). However, the positive predictive value was only 48.2% (95% CI 35.7 to 61.0%) and negative predictive value was 72.6% (95% CI 60.4 to 82.1%).

## Discussion

Multiple studies in patients with a spectrum of intravascular volume ranging from ALI to CHF indicate that the VPW measured from a CXR correlates highly with intravascular pressure and distinguishes cardiogenic from non-cardiogenic edema, but this is the first study to our knowledge assessing the role of this easily measured anatomic landmark among patients exclusively with ALI (a markedly narrower intravascular volume range). VPW correlated moderately well with PAOP and less well with CVP. In multivariate regression, the correlation between VPW and PAOP was stronger than that between net cumulative fluid balance or PEEP and PAOP, while serum albumin did not independently correlate with PAOP. Furthermore, VPW decreased over time in the conservative fluid management strategy arm, but increased in the liberal fluid management arm. VPW, however, was only moderately able to discriminate achievement of the conservative fluid management target of PAOP <8 mmHg and unable to discriminate achievement of CVP <4 mm Hg. VPW was also only moderately able to discriminate whether a hydrostatic component of the edema may also be present in these patients with ALI. These new observations provide additional data on the reliability and clinical relevance of this non-invasive radiologic measurement.

Although underutilized, determining intravascular volume status by radiographic appearance has classically revolved around measurement of the VPW and analysis of patterns of lung parenchymal infiltration [8,13,14]. A review of acute pulmonary edema recommended the VPW as a potentially useful factor in differentiating cardiogenic from non-cardiogenic pulmonary edema [15]. Initially characterized in upright posteroanterior CXRs from non-critically ill patients, the VPW measurement has subsequently been shown to have similar predictive ability in ICU patients with anteroposterior supine films [6,9,10]. Several investigations have addressed relationships between VPW and intravascular volume status [12,16,17]. Other studies have demonstrated the ability of the VPW to differentiate pulmonary edema due to volume overload from that due to acute lung injury [6,9,10]. Our optimal cutoff of a VPW ≥72 mm for distinguishing a hydrostatic component to the pulmonary edema was similar to the values of 68 and 70 mm found in previous studies [6,9]. In addition to confirming the findings of these studies, our data also suggest that VPW might be able to be used to identify when hydrostatic edema may be contributing to ALI and whether conservative fluid management targets have been reached in cases where intravascular pressure measurements are not available.

Application of VPW measurement or the necessity for uptake into clinical practice has been marginal because of the decreasing prevalence of placement of invasive catheters such as pulmonary artery or central venous catheters as well as unfamiliarity with data related to its measurement and potential value when invasive tools are not in place. In the current period of critical care in which fewer pulmonary artery catheters are placed, most intravascular measurements are taken on a routine basis from the conventional catheter measuring a CVP. Of note, in this investigation, VPW correlated with PAOP better than CVP.

It is helpful to be facile with factors that can increase or reduce the VPW. The supine position can increase the VPW by nearly 20% compared to the upright position [5], and thus the "normal" VPW on films taken when the patient is supine would be 58 to 62 mm. Rotation of the patient to the right artificially increases the VPW, while rotation to the left decreases the measurement [11]. Importantly, in this study all the patients' CXRs and intravascular measurements were taken in the supine or semi-supine position and only films graded as satisfactory for positioning (that is, not overly rotated on visual inspection) were included in the analysis. In addition to patient positioning, some have raised concern that the disease process might affect the assessment of VPW. Indeed, the effects of recent trauma, thoracic surgery, or prior radiation therapy alter components of the mediastinal silhouette and compromise the utility of the VPW [18,19]. On the other hand, respiratory factors have been shown to have relatively little effect on VPW measurements. Milne observed comparable VPW measurements during both inspiration and expiration [5]. Although mechanical ventilation may have profound effects upon other radiographic findings such as the pattern and severity of parenchymal infiltrates [20,21], VPW measurements have been found to be consistent between spontaneous and positive pressure breaths [20]. Our data also found only a trend toward a weak correlation between PEEP and VPW measurements. Despite these potential limitations in measuring the VPW, we confirmed prior findings that VPW correlates with PAOP and we found that the VPW correlated 1.5 times better with PAOP than cumulative net fluid balance and 2.5 times better than PEEP. Thus, for patients without or for clinicians who prefer not to use invasive intravascular pressure measurements, VPW represents a better surrogate of PAOP than net fluid balance.

One limitation of our study is that we compare VPW to two surrogate measures of intravascular volume, CVP and PAOP, and not a direct measure of intravascular volume, such as right (RVEDV) or left ventricular end-diastolic volume (LVEDV). Although echocardiography might estimate RVEDP and LVEDP, too few patients had these available on days with VPW measurements to investigate this correlation directly. CVP and PAOP do correlate well with right (RVEDP) and left ventricular end-diastolic pressure (LVEDP), respectively [22-24]. Although a similar correlation with RVEDV and LVEDV is widely presumed, this is not the case in a number of conditions pertinent to acute lung injury, including sepsis [25-27], trauma [28], and acute respiratory failure requiring mechanical ventilation [29]. Observations by Kumar and colleagues suggest that CVP and PAOP do not correlate well with RVEDV or LVEDV even in normal, healthy volunteers [30]. This is likely due to varying compliance of the ventricles from patient to patient and heartbeat to heartbeat within the same patient. Because VPW is an objective, anatomic measurement of vascular structures, it is likely influenced less than CVP and PAOP by outside forces such as mechanical ventilation, PEEP, large intrathoracic pressure variations during the respiratory cycle, and even varying cardiac compliances. As such, VPW may prove to be a more accurate measure of intravascular volume than either CVP or PAOP and may correlate better with actual intravascular volume than these intravascular pressure surrogates. Although our data lack a direct intravascular volume measurement, future studies could incorporate one as a different reference standard. It is noteworthy that even in this selected population of patients with noncardiogenic pulmonary edema, that VPW measurements moderately differentiated volume status.

**Figure 5:**
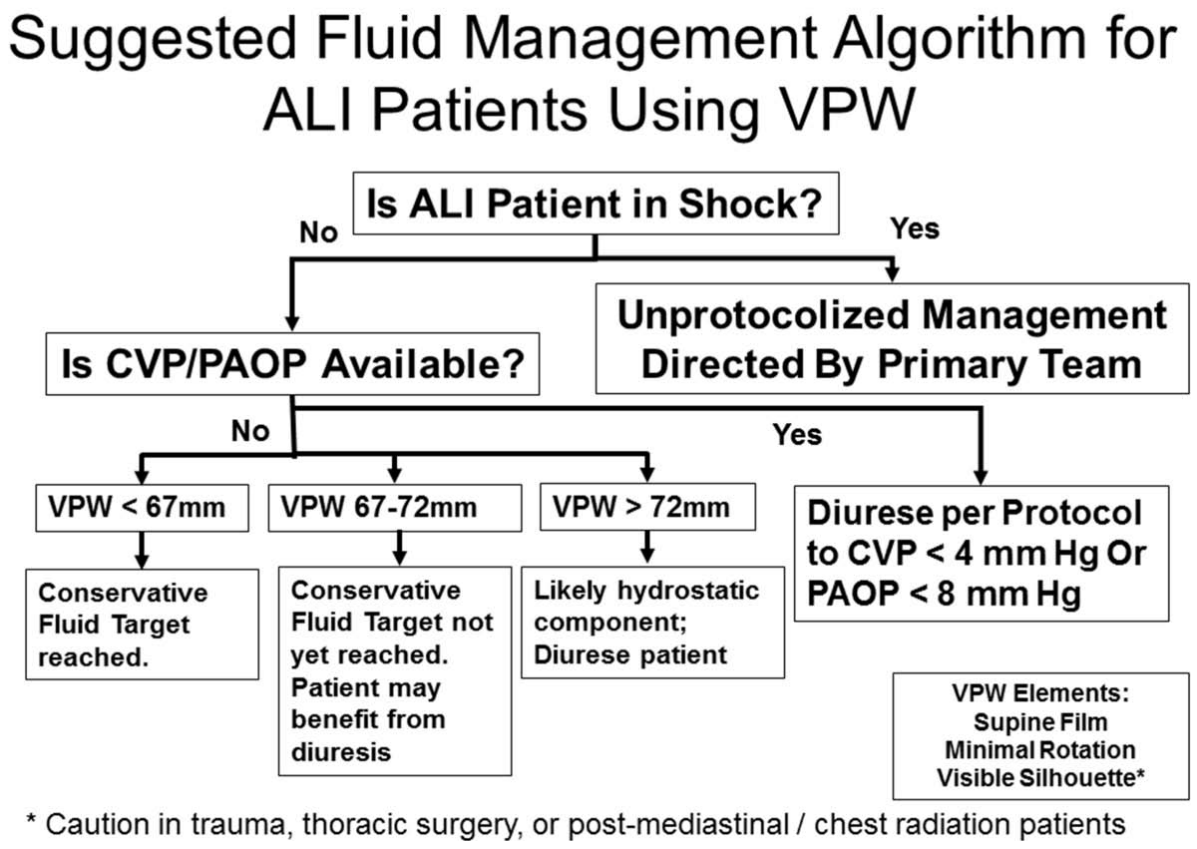
**Suggested fluid management algorithm for ALI patients using VPW**.

Our study also has other limitations. The patients enrolled in FACTT are a highly-selected group of patients with acute lung injury. This substudy evaluates data from a subset of the overall FACTT population. However, almost 30% of the enrolled patients were included, with five geographically diverse centers with heterogeneous patient populations participating. Although all the data were collected prospectively during the conduct of the original study, this substudy represents a *post-hoc*, retrospective analysis. As such, many of the CXR and vascular pressure measurements did not occur simultaneously. To minimize any potential bias this might introduce, we limited our analysis to "matched" measurements and CXRs obtained within three hours of each other. Furthermore, although a VPW of 67 mm, was found to best predict a PAOP <8 mmHg the relatively few instances that conservative fluid management resulted in target PAOP or CVP measurements being reached resulted in wide confidence intervals for the sensitivity and specificity. Similar to the cutoffs previously defined for differentiating patients with cardiogenic versus noncardiogenic edema [6,9], a VPW value of 72 or higher in our study, also discriminated a PAOP of at least 18 mmHg, which could represent cases where volume overload and hydrostatic edema may be contributing to the hypoxia and patients who may benefit from diuresis. Despite only having moderate sensitivity and specificity for predicting either volume overload or conservative fluid status, given its non-invasive nature, relative availability, and moderate sensitivity and sensitivity, we think these data support the use of VPW in a fluid management strategy when other measures, such as intravascular pressure measurements, are unavailable. A suggested algorithm is presented in Figure 5.

This study also has a number of strengths. We averaged the VPW measurements from multiple, independent, blinded readers of the CXRs, ranging from a seasoned radiologist to intensivists with both extensive and limited prior experience in measuring VPW. Although inter-rater variability in this study was higher than that seen in previous studies [6,10], the VPW was still a significant predictor of intravascular status of the cohort. This variability, likely secondary to the number of readers and inexperience of two readers, might be reduced through standardized teaching and more experience, yielding even more striking results. Despite the relatively small number of patients, ours still represents one of the largest studies of VPW measurements to date. In addition to confirming a relationship between VPW and intravascular pressure measurements, this investigation also introduces the novel idea that VPW can be used to identify when conservative fluid management targets have been reached. The nature of the data collected allowed us to compare VPW with both PAOP and CVP and to compare the effect of other possible confounders, such as cumulative fluid balance, PEEP, and serum albumin on the relationship.

The FACTT study demonstrated that patients with ALI treated with a conservative fluid strategy had significantly more days alive and free from mechanical ventilation and alive and out of the ICU compared to those managed with a more liberal fluid management strategy [4]. Despite these important outcome benefits, widespread implementation of a conservative fluid strategy in practice has been relatively slow [31]. The reasons for this delayed acceptance are likely multifactorial, including lack of survival benefit and the relative complexity of the management algorithm, which includes the need for some assessment of intravascular pressure. Invasive measurements were utilized in the clinical trial, with similar outcomes resulting from CVP and PAOP measurements [1]. While this likely will contribute to a further reduction in the insertion of PACs, obtaining CVP measurements still requires an invasive procedure and risk for complications. Although many patients with ALI have central venous catheters placed for routine care, the frequency of invasive procedures is decreasing in clinical practice and 8.1% of patients were excluded from the parent study due to physicians not intending to place central venous access [1]. The ability to utilize non-invasive measures of intravascular volume may obviate the need for a CVC in some patients and further reduce the risk of complications. The use of the non-invasive VPW may enhance implementation and acceptance of the conservative fluid strategy into routine clinical practice. It remains to be established whether fluid adjustments made on the basis of VPW measurements achieve similar outcomes as strategies guided by invasive hemodynamic measurements.

## Conclusions

VPW correlated moderately well with PAOP and less well with CVP in patients with ALI enrolled in a clinical trial of different fluid management strategies. VPW had a higher correlation with the historical standard of PAOP than did cumulative fluid balance or PEEP. Although the actual correlation between VPW and direct intravascular volume measurements remains unknown, these data confirm previous studies that show the utility of VPW as a noninvasive measure and the best radiographic sign of patients' intravascular volume status. VPW is measured easily on most CXRs and might be useful for discriminating when a hydrostatic component of the edema may be contributing or conservative fluid management pressure targets have yet to be reached in patients with ALI when invasive vascular pressure measurements are unavailable. Routine substitution of VPW for CVP or PAOP in fluid management of ALI patients cannot be recommended, however, until a trial using VPW directly to titrate diuretic dosing has been completed.

## Key messages

• In ventilated ICU cohorts of both high and low intravascular volume status (for example, ALI and CHF), the VPW has been consistently shown as a correlate of intravascular volume status.

• In this study restricted to ALI patients, the "non-invasively obtained" VPW correlated with PAOP better than CVP.

• Changes in VPW correlated with changes in volume status.

• VPW had a 1.5-fold stronger correlation with PAOP than cumulative fluid balance and a 2.5-fold stronger correlation than PEEP.

• Within the narrower range of volume status presented by restricting this cohort to only ALI, the ability of VPW to discriminate a hydrostatic component of the edema and achievement of fluid management goals was limited.

• Given its non-invasive nature and availability, VPW might still be able to be used to direct fluid management in patients with ALI when intravascular pressure measurements are unavailable.

## Abbreviations

ALI: acute lung injury; ARDS: acute respiratory distress syndrome; AUC: area under the curve; CTR: cardiothoracic ratio; CVC: central venous catheter; CVP:central venous pressure; CXR: chest X-ray; FACTT: Fluid and Catheter Treatment Trial; ICU: intensive care unit; IQR: interquartile range; IRB: institutional review board; LVEDP: left ventricular end-diastolic pressure; LVEDV: left ventricular end-diastolic volume; NHLBI: National Heart Lung and Blood Institute; NIH: National Institutes of Health; PAC: pulmonary artery catheter; PAOP: pulmonary artery occlusion pressure; PEEP: positive end-expiratory pressure; ROC: receiver operating characteristic; RVEDP: right ventricular end-diastolic pressure; RVEDV: right ventricular end-diastolic volume; VPW: vascular pedicle width; 95% CI: 95% confidence interval.

## Competing interests

The authors declare that they have no competing interests.

## Authors' contributions

All authors participated in the design of the study and data acquisition. TWR, LBW, EWE, CC and EH interpreted the CXRs. TWR, EWE and LBW analyzed and interpreted the data. TWR, EWE and LBW drafted the manuscript. EWE, LBW, MAM, RDH, JSS and EH revised the manuscript critically for important intellectual content. All authors read and approved the final manuscript.
